# De-escalation of antiplatelet therapy in acute coronary syndromes: Why, how and when?

**DOI:** 10.3389/fcvm.2022.975969

**Published:** 2022-08-25

**Authors:** Mattia Galli, Dominick J. Angiolillo

**Affiliations:** ^1^Catholic University of the Sacred Heart, Rome, Italy; ^2^Maria Cecilia Hospital, GVM Care and Research, Cotignola, Italy; ^3^Division of Cardiology, University of Florida College of Medicine, Jacksonville, FL, United States

**Keywords:** de-escalation, antiplatelet therapy, percutaneous coronary intervention, acute coronary syndrome, dual antiplatelet therapy

## Abstract

The synergistic blockade of the key platelet signaling pathways of cyclooxygenase-1 blockade and P2Y_12_ signaling by combining aspirin plus a potent P2Y_12_ inhibitor (prasugrel or ticagrelor), the so called dual antiplatelet treatment (DAPT), has represented the antithrombotic regimen of choice in patients with acute coronary syndrome (ACS) for nearly a decade. Nevertheless, the use of such antiplatelet treatment regimen, while reduced the risk of thrombotic complications, it is inevitably associated with increased bleeding and this risk may outweigh the benefit of a reduction of ischemic events in specific subgroup of patients. In light of the adverse prognostic implications of a bleeding complication, there has been a great interest in the development of antiplatelet regimens aimed at reducing bleeding without any trade-off in ischemic events. The fact that the ischemic risk is highest in the early phase after an ACS while the risk of bleeding remains relatively stable over time has represented the rationale for the implementation of a more intense antithrombotic regimen early after an ACS, followed by a less intense antithrombotic regimen thereafter. This practice, known as a “de-escalation” strategy, represents one of the more promising approaches for personalization of antithrombotic therapy in ACS. In this review we discuss the rationale, appraise the evidence and provide practical recommendations on the use of a de-escalation strategy of antiplatelet therapy in patients with an ACS.

## Introduction

Dual antiplatelet treatment (DAPT), consisting in the combination of aspirin and a P2Y_12_ inhibitor, represents the antithrombotic regimen of choice for the prevention of thrombotic complications in patients with acute coronary syndrome (ACS) as well as for those undergoing percutaneous coronary intervention (PCI) (1). Indeed, the synergistic effects of blocking the key platelet signaling pathways of cyclooxygenase-1 blockade and P2Y_12_ signaling has been associated with elevated antithrombotic efficacy coupled with a relatively favorable safety profile (2, 3). Moreover, the more predictable and potent P2Y_12_ inhibitors prasugrel and ticagrelor have shown to reduce ischemic events at the cost of increased bleeding compared with clopidogrel (4, 5). Therefore, in the absence of contraindications, a period of 12-month DAPT using a potent P2Y_12_ inhibitor represents the standard-of-care in ACS (6, 7). Nevertheless, because the use of this antiplatelet regimen is inevitably associated with increased bleeding risk and this risk may outweigh the benefit of a reduction of ischemic events in specific subgroup of patients, there has been a great interest in the personalization of antithrombotic therapy over the past years (8). This is further emphasized by the fact that a number of studies have demonstrated the adverse prognostic implications, including increased mortality, associated with a bleeding complication (9). Current guidelines, have proposed personalization of antiplatelet therapy according to patient’s bleeding and ischemic risk, with dedicated scores designed to predict and standardize such risks (6, 7). Nevertheless, in the real-world setting the use of scores is limited by several challenges such as the large overlap between factors associated with increased ischemic and bleeding events. Moreover, current recommendations does not take into account the important interindividual variability in response to P2Y_12_ inhibitors, which is strongly associated with adverse events (10, 11). The fact the ischemic risk is highest in the early phase after ACS while the risk of bleeding remains relatively stable over time has represented the rationale for the implementation of a more intense antithrombotic regimen early after an ACS, followed by a less intense antithrombotic regimen thereafter, in the attempt to reduce bleeding without any trade-off in ischemic events (1). Such approach, known as “de-escalation” strategy, represents one of the more promising approaches for personalization of antithrombotic therapy in ACS. In this review we discuss the rationale, appraise the evidence and provide practical recommendations on the use of such strategy.

## De-escalation: Why?

For many years, the main focus of antithrombotic therapy in patients with ACS or undergoing PCI has been the reduction of ischemic events, including both ischemic recurrences, such as spontaneous myocardial infarction (MI), and local ischemic events, such as stent thrombosis (ST) (12). In particular, a more prolonged and intense antithrombotic therapy has been implemented in the attempt of preventing ST, a much feared complication of PCI associated with poor prognosis, whose incidence was relatively more frequent with the advent of first generation drug eluting stent (DES) (13). Conversely, from the past decade, a particular attention has been paid to the most relevant adverse event associated with antithrombotic therapy: bleeding (9, 14). Indeed, with the advent of second and third generation DES, the incidence of ST, including those occurring late and very late after PCI, has dramatically reduced, making prolonged and intense antithrombotic therapies no longer necessary for this purpose (13). Furthermore, there has been an increased understanding of the prognostic impact of a bleeding complication among patients with ACS or those undergoing PCI, which, in some cases, can be associated with a similar or even worse prognosis compared with ischemic events such as a spontaneous MI (15). Finally, it is now well known that the incidence of both local and ischemic events is highest during the first months after PCI and tends to decrease thereafter (1). On the contrary, the risk of bleeding, despite being relatively greater in the first days after PCI mainly because of the use of an arterial access site and periprocedural antithrombotic therapy, remains relatively stable over time (1, 9). Therefore, a strategy of more intense antithrombotic therapy in the first 1–3 months after ACS/PCI followed by de-escalation of antiplatelet therapy thereafter has been implemented in several recent randomized controlled trials (RCTs), showing very promising results.

Further evidence in support of a de-escalation strategy of antiplatelet therapy comes from studies showing that the use of prasugrel or ticagrelor is not associated with reduced ischemic events among patients “responders” to clopidogrel, but, on the contrary, greater platelet inhibition provided by prasugrel and ticagrelor more often results in low platelet reactivity (LPR), which has been associated with increased risk of bleeding without any reduction of ischemic events among patients responding to clopidogrel (10, 16). Indeed, clopidogrel, but not prasugrel or ticagrelor, is associated with high platelet reactivity (HPR), a marker of thrombotic risk, in 20–40% of treated patients (the so called clopidogrel “poor responders”) (10, 17–19). This interindividual variability in clopidogrel response stems from the fact that clopidogrel is a pro-drug that requires a two-step oxidation process by the hepatic cytochrome P450 (CYP) 2C19 system to be activated, but the gene responsible for the transcription of such enzyme is highly polymorphic, with patients who are carriers of CYP2C19*2 and CYP2C19*3 (loss-of-function, LoF, alleles) being associated with diminished enzyme activity (17, 20). The distribution of *CYP2C19* alleles allows for defining 5 different phenotypes: ultrarapid (UM), rapid (RM), normal (NM), intermediate (IM), and poor (PM) metabolizers (17, 21). PM are carriers of 2 LoF *CYP2C19* alleles (e.g., *2/*2 or *2/*3 genotype) and IM are carriers one LoF allele (18), both (particularly the former) are associated with reduced levels of clopidogrel active metabolite, HPR and increased thrombotic risk (17, 20). Notably, there are other clinical risk factors that can contribute to HPR, including age, body mass index, chronic kidney disease, and diabetes mellitus (22). Overall, such evidence represents the rationale for a guided de-escalation strategy of P2Y_12_ inhibiting therapy from prasugrel or ticagrelor to clopidogrel selectively among patients found to adequately respond to clopidogrel, with the aim of decreasing bleeding without any trade-off in ischemic events.

## De-escalation: How?

**FIGURE 1:**
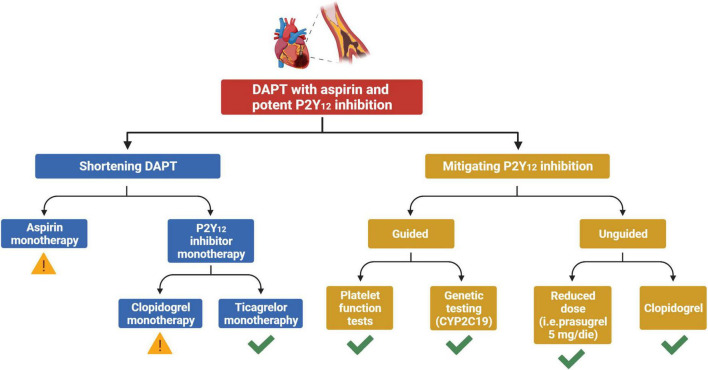
Strategies for a de-escalation of antiplatelet therapy among patients with ACS undergoing PCI. Yellow exclamation mark, strategy associated with possible increased ischemic risk. Green check, strategy not associated with any trade-off in ischemic events. ACS, acute coronary syndrome; DAPT, dual antiplatelet therapy; CYP, cytochrome P450.

A de-escalation strategy of antiplatelet therapy in ACS aims at reducing bleeding without any trade-off in ischemic events. The two main de-escalation approaches currently adopted in patients with ACS consist in the shortening of DAPT duration and in the mitigation of P2Y_12_ inhibition after a short course of standard DAPT. The former may be classified according to the single antiplatelet agent used after shortening of DAPT (aspirin vs. P2Y_12_ inhibitor monotherapy), while the latter strategy may be classified depending on whether tools to guide the selection of P2Y_12_ inhibition are used or not (guided vs. unguided de-escalation) (Figure 1). The main features and pitfalls of RCTs testing a de-escalation of antiplatelet therapy among ACS patients are summarized in Table 1. Although the comparative effects of different de-escalation strategies seem to favor the mitigation of P2Y_12_ inhibition for efficacy and short DAPT for safety, these findings are mostly based on indirect comparisons and require further investigations (23).

**TABLE 1:** Randomized controlled trials testing antiplatelet de-escalation strategies in patients with acute coronary syndrome undergoing PCI.

Study name	Number of patients enrolled	Timing of de-escalation	Primary endpoint	Limitations	Follow-up duration
**Shortening DAPT**					
**Aspirin monotherapy**					
DAPT-STEMI (2018)	1,100	6 months	All death, MI, any revascularization, stroke, and TIMI major bleeding	Non-inferiority design Primary endpoint including both ischemic and bleeding outcomes	18 months
SMART-DATE (2018)	2,712	6 months	All death, MI or stroke	Non-inferiority design Short DAPT was associated with a doubled risk of MI and with a 50% increased risk of ST East Asian population	18 months
REDUCE (2019)	1,496	3 months	All death, MI, ST, stroke, target vessel revascularization and BARC 2–5 bleeding	Non-inferiority design Primary endpoint including both ischemic and bleeding outcomes Short DAPT was associated with a doubled risk of ST and a 62% increased risk of CV death	12 months
**Clopidogrel monotherapy**					
STOPDAPT-2-ACS (2022)	4,169	1–2 months	CV death, MI, stroke, ST and TIMI major or minor bleeding	Non-inferiority design Primary endpoint not met Primary endpoint including both ischemic and bleeding outcomes Short DAPT was associated with a 50% increase in the composite of CV death, MI, ST and stroke and a nearly doubled risk of MI East Asian population	12 months
**Ticagrelor monotherapy**					
GLOBAL-LEADERS (ACS sub-study)	3,750	1 month	All death or MI	Sub-study of a RCT	24 months
TWILIGHT (ACS sub-study)	4,614	3 months	All death or MI and BARC 2–5 bleeding	Sub-study of a RCT Primary endpoint including both ischemic and bleeding outcomes Randomization limited to uneventful patients after 3 months of standard DAPT	15 months
TICO (2020)	3,056	3 months	TIMI major bleeding, all-cause death, MI, ST, stroke, and target-vessel revascularization	Primary endpoint including both ischemic and bleeding outcomes Low ischemic risk patients East Asian population	12 months
**Mitigating P2Y_12_ inhibition**					
**Guided**					
**Platelet function-guided**					
ANTARCTIC (2016)	877	14 days (and 28 days)	CV death, MI, stroke, ST, urgent revascularization and BARC 2–5 bleeding	Primary endpoint not met Primary endpoint including both ischemic and bleeding outcomes Use of prasugrel 5 mg rather than prasugrel 10 mg Randomization 14 days after ACS	12 months
TROPICAL-ACS (2017)	2,610	7 days (and 14 days)	CV death, MI, stroke, and BARC 2–5 bleeding	Non-inferiority design Primary endpoint including both ischemic and bleeding outcomes	12 months
***Genotype-guided***					
POPular Genetics (2019)	2,488	< 2 days	Death from any cause, MI, definite ST, stroke, or major bleeding defined according to PLATO criteria and PLATO major or minor bleeding	Non-inferiority design Primary endpoint including both ischemic and bleeding outcomes	12 months
**Unguided**					
***Unguided clopidogrel***					
TOPIC (2017)	646	1 month	CV death, urgent revascularization, stroke and BARC 2–5 bleeding	Non-inferiority design Primary endpoint including both ischemic and bleeding outcomes Relatively small trial	12 months
TALOS-MI (2021)	2,697	1 month	CV death, MI, stroke and BARC 2–5 bleeding	Non-inferiority design Primary endpoint including both ischemic and bleeding outcomes East Asian population	12 months
***Reduced dose of P2Y_12_ inhibitor***					
HOST-REDUCE-POLYTHEC-ACS (2020)	3,429	1 month	All death, MI, ST, repeat revascularization, stroke and BARC 2–5 bleeding	Non-inferiority design Primary endpoint including both ischemic and bleeding outcomes East Asian population	12 months

ACS, acute coronary syndrome; DAPT, dual antiplatelet therapy; TIMI, Thrombolysis in Myocardial Infarction; MI, myocardial infarction; CV, cardiovascular; ST, stent thrombosis; PFT, platelet function test; BARC, Bleeding Academic Research Consortium; PLATO, Platelet Inhibition and Patient Outcomes; RCT, randomized controlled trial; PCI, percutaneous coronary intervention.

### Shortening dual antiplatelet treatment

The shortening of DAPT duration has been tested for the first time a decade ago and has been the most broadly explored de-escalation strategy over years (1). The shortening of DAPT typically consists in the withdrawal of the P2Y_12_ inhibitor before the recommended standard period of DAPT, usually 3 or 6 months after PCI. More recently, the discontinuation of aspirin and the maintaining of P2Y_12_ inhibitor monotherapy 1 or 3-month after ACS or PCI has been tested and also implemented in practice guidelines.

### Shortening dual antiplatelet treatment by stopping a P2Y_12_ inhibitor and maintaining aspirin

From 2012 to the present day, thirteen studies have tested a shortening of DAPT duration followed by aspirin monotherapy among patients undergoing PCI, but only three focused on patients with ACS (1). Although the majority of RCTs, as well as several pooled analyses, showed a very short DAPT followed by clopidogrel monotherapy may reduce bleeding without any trade-off in ischemic events, a particular attention should be paid on the three RCTs selectively enrolling ACS patients (24, 25). Among these, DAPT-STEMI and SMART-DATE compared a 6-month DAPT and REDUCE compared a 3-month DAPT, vs. standard 12-month DAPT (26–28). Despite all these three trials met their primary endpoint, all of them had non-inferiority designs and two of them had a primary endpoint including both ischemic and bleeding events (26–28). Because the main concern when de-escalating antithrombotic therapy is the possible trade-off in ischemic events while a reduction of bleeding is expected, such designs do not completely reassure on the use of such strategy, especially in the light of several pitfalls shown by these studies (Table 1). In particular, short DAPT was associated with a twofold increased risk of MI and a 50% increased risk of ST in the SMART-DATE trial and with a twofold increased risk of ST and a 62% increased risk of CV death in the REDUCE trial (27, 28). Furthermore, an individual patient data meta-analysis comparing short (either 3 or 6 month) vs. standard 12-month DAPT duration in 11,473 patients stratified according to clinical presentation (CCS *n* = 6,714; ACS *n* = 4,758) provided important insights to this extent. In ACS,?but not in CCS patients, 3-month DAPT was associated with higher rates of MI or ST and ≤ 6-month DAPT was associated with a trend toward increased rates of MI or ST at 1 year compared with 12-month DAPT (29).

### Shortening dual antiplatelet treatment by stopping aspirin and maintaining clopidogrel

Two RCTs provided encouraging results on the safety and efficacy of a short (1 or 3-month) DAPT followed by clopidogrel monotherapy among mixed populations of ACS and CCS undergoing PCI (30, 31). Nevertheless, the recently published STOPDAPT-2-ACS non-inferiority trial, which focused on ACS patients, underlined important limitations of such strategy (32). STOPDAPT-2-ACS randomized patients to either a 1–2 months of DAPT followed by clopidogrel monotherapy (*n* = 2,078) or to 12 months of DAPT with aspirin and clopidogrel (*n* = 2,091). The primary end point was a composite of CV death, MI, any stroke, or definite ST or bleeding (Thrombolysis in MI major or minor bleeding, TIMI) events at 1 year, with a non-inferiority margin of 50%. One to 2 months of DAPT was not non-inferior to 12 months of DAPT for the primary end point (3.2% vs. 2.8%; HR 1.14; 95% CI 0.80–1.62). Moreover, the major secondary ischemic endpoint of CV death, MI, any stroke, or definite ST was borderline significantly increased in the short compared with standard DAPT arm (2.8% vs. 1.9%; HR 1.50; 95% CI 0.99–2.26) (32). These findings call for caution for such an early drop of aspirin among ACS patients undergoing unguided use of clopidogrel monotherapy. It is important to note that all the RCTs conducted in this setting, including STOPDAPT-2-ACS enrolled East Asians, a population with lower ischemic and greater bleeding events compared to other ethnicities (33). Therefore such an increase of ischemic events may underestimate the potential for ischemic events in Westerners and requires further investigations among populations of different ethnicities.

### Shortening dual antiplatelet treatment by stopping aspirin and maintaining prasugrel

To date, prasugrel monotherapy after a short course of DAPT was tested only in a pilot study including 201 patients with CCS undergoing low-risk PCI (34). No evidence is available in ACS patients which is a topic of ongoing investigation (NCT04360720).

### Shortening dual antiplatelet treatment by stopping aspirin and maintaining ticagrelor

As opposed to a strategy of clopidogrel monotherapy, a strategy of ticagrelor monotherapy after 1–3 months of DAPT has been shown to be safe and effective in both RCTs including ACS and CCS patients and those focusing on ACS (35, 36). The TICO trial randomized 3,056 ACS patients from South Korea to receive ticagrelor monotherapy after 3-month DAPT or ticagrelor-based 12-month DAPT (36). The primary outcome was a composite of death, MI, ST stroke, target-vessel revascularization or TIMI major bleeding. At 1 year, ticagrelor monotherapy after 3-month DAPT was associated with reduced primary endpoint (3.9% vs. 5.9%; HR 0.66; 95% CI 0.48–0.92) compared with ticagrelor-based 12-month DAPT. The observed benefit was driven by a reduction of major bleeding (1.7% vs. 3%; HR 0.56; 95% CI 0.34–0.91) but also by a trend toward reduced composite ischemic events (2.3% vs. 3.4%; HR 0.69; 95% CI 0.45–1.06) with ticagrelor monotherapy (36). A significant reduction of major bleeding paralleled by no trade-off in ischemic events was also observed in the pre-specified sub-analysis of GLOBAL-LEADERS and TWILIGHT according to clinical presentation (ACS vs. CCS) (37). Indeed, the reduction of bleeding was greater in ACS as compared to CCS patients in these pre-specified analyses, suggesting ACS patients are those who could benefit the most from such strategy (37, 38). Collectively, the data in support of the use of ticagrelor monotherapy after one or 3 month-DAPT among ACS are encouraging and comes from evidence from both Eastern and Western countries.

### Modulating P2Y_12_ inhibition

Modulation of P2Y_12_ inhibition, achieved by using less potent or reduced dose of P2Y_12_ inhibitors, may be either guided or unguided, depending on the use or not of specific tools aiming at implementing a personalized approach that could take into account the interindividual variability in response to P2Y_12_ inhibitors.

### Guided approach

**FIGURE 2:**
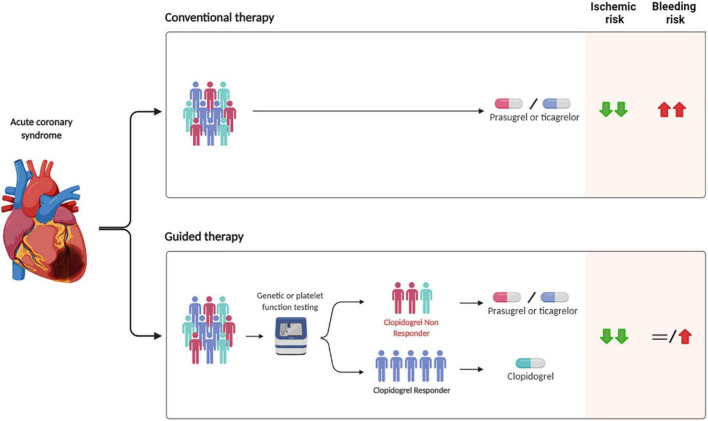
Safety and efficacy of guided vs. conventional selection of P2Y_12_ inhibitors in patients with ACS undergoing PCI. PCI, percutaneous coronary intervention.

Two different tools can be used to allow for a guided de-escalation of antiplatelet therapy in clinical practice: platelet function (PFT) and genetic tests (17, 18, 39) (Figure 2). PFT can be either laboratory-based or point-of-care, with the latter being preferred over the former for practical reasons, including ease of use and for providing results in a timely fashion, while genetic testing allows for the identification of genetic variants, including LoF alleles, of the *CYP2C19* gene (17, 39). Each test has advantages and disadvantages. PFT presents the fundamental advantage of directly identifying phenotypes (i.e., levels of platelet reactivity) associated with increased thrombotic (i.e., HPR) or bleeding (i.e., LPR) risk, but has inherent limitations including the need for multiple assessments due to possible variability of results over time and the need for a patient to be on treatment with a given antiplatelet agent to define responsiveness (17, 40, 41). This latter issue is particularly problematic in the setting of ACS in which prasugrel and ticagrelor represent the standard of care, and would require a patient to switch to clopidogrel and be on maintenance therapy for at least 7–14 days in order to best assess drug response. Genetic testing for *CYP2C19* alleles has the key advantage that the genetic makeup of an individual remains unvaried and does not require a patient to be on treatment with clopidogrel, but presents the disadvantage that *CYP2C19* genotypes represents only one of the factors contributing to clopidogrel response. Integrating genetic data with clinical variables (age, body mass index, chronic kidney disease, and diabetes mellitus) such as in the ABCD-GENE score can enhance the accuracy in identifying individual with impaired clopidogrel response (i.e., HPR status) (22, 41, 42). Importantly, the implementation of these tools is associated with reduced costs due to the larger use of generic P2Y_12_ inhibitor formulations and reduced clinical events and hospitalizations (43).

Two studies have tested a strategy of PFT-guided de-escalation among ACS patients. The first, ANTARCTIC, randomized 877 elderly patients with ACS undergoing PCI to receive prasugrel 5 mg daily with dose or drug adjustment in case of inadequate response (monitoring group) or oral prasugrel 5 mg daily with no monitoring or treatment adjustment (conventional group) (44). PFT was done 14 days after randomization and repeated 14 days after treatment adjustment in patients in the monitoring group. There was no difference in the primary endpoint of net adverse clinical events (NACE) between groups (44). A major limitation of this trial potentially blunting the superior safety of a de-escalation strategy is that prasugrel 5 mg daily was used in lieu of the standard dosage of 10 mg daily (44). The second RCTs in this setting was the larger TROPICAL-ACS that randomized 2610 ACS patients to either standard treatment with prasugrel 10 mg for 12 months (control group) or a step-down regimen (1 week prasugrel followed by 1 week clopidogrel and PFT-guided maintenance therapy with clopidogrel or prasugrel 10 mg from day 14 after hospital discharge; guided de-escalation group) (45). The study met the primary non-inferiority endpoint of net clinical benefit including CV death, MI, stroke or BARC bleeding 2–5 at 1 year with a margin of 30% (45). Furthermore, there was no increase in the combined risk of CV death, MI, or stroke but a trend toward lower bleeding in the de-escalation group (45). The main limitation of RCTs testing a PFT-guided de-escalation is represented by the delay in the initiation of a guided therapy due to the need for a patient to switch to clopidogrel before PFT can be performed. Indeed, patients need to be switched to clopidogrel for the test to be performed and back to a potent P2Y_12_ inhibitor in case of HPR while on clopidogrel.

With regards to a genotype-guided de-escalation strategy, POPular Genetics randomized 2,488 STEMI patients to either genotype-guided de-escalation or standard therapy (mainly ticagrelor) within 48 h after PCI (46). The trial met both the non-inferiority primary endpoint of NACE and found a significant 22% reduction of the co-primary endpoint of Platelet Inhibition and Patient Outcomes (PLATO) major and minor bleeding at 12 months among (46). Importantly, there was no trade-off in ischemic events, which were on the contrary numerically reduced in the guided as compared to standard therapy arm (2.7% vs. 3.3% for the combined composite endpoint of CV death, MI, ST or stroke) (46). Finally, TAILOR PCI, the largest trial comparing guided vs. standard antiplatelet therapy, included both ACS (69%) and CCS (31%) and implemented a strategy of escalation rather than de-escalation of antiplatelet therapy, providing limited evidence on this latter strategy in ACS (16, 43).

It may be argued that because the more relevant studies on guided de-escalation (i.e., TROPICAL-ACS and POPular Genetics) used a primary composite endpoint including both ischemic and bleeding outcomes and had non-inferiority designs, their statistical power is limited with respect to hard ischemic (i.e., CV death, MI, ST) and bleeding events (i.e., major bleeding and intracranial hemorrhage). Nevertheless, a recent comprehensive meta-analysis improving statistical power for such outcomes showed that a strategy of guided de-escalation is associated with a 19% reduction of bleeding without any trade-off in ischemic events (47). Furthermore, a network meta-analysis focusing on ACS showed that a guided selection of P2Y_12_ inhibitors was associated with superior safety and effectiveness as compared with prasugrel or ticagrelor (Figure 2) (19).

### Unguided approach

Based on the premise that the early weeks after ACS are characterized by a higher thrombotic risk that could benefit the most from an aggressive antiplatelet therapy while in the months following an acute coronary event the bleeding risk may run higher than the thrombotic risk, a proposed practical approach has been that of simply awaiting for the highest risk period of thrombotic complications post-PCI to elapse (e.g., 1 month) prior to de-escalation. Of note, an unguided de-escalation early after ACS has been associated with an increase in thrombotic complications which can be attributed to the high platelet reactivity after ACS as well as drug interactions when switching from a P2Y_12_ inhibitor to another (i.e., from ticagrelor to clopidogrel), and should be strongly discouraged (48, 49). Two RCTs have tested a standard vs. unguided de-escalation from a potent platelet inhibitor (i.e., prasugrel or ticagrelor) to clopidogrel and one RCT has tested a standard vs. unguided de-escalation from full dose (10 mg daily) to reduced dose (5 mg daily) of prasugrel, 1 month after ACS (50–52). Despite several limitations of these trials such as the non-inferiority designs, low-risk PCI, and the inclusion of primary endpoints including both ischemic and bleeding events (Table 1), all met their primary endpoint and a recent meta-analysis pooling evidence from these three trial has shown that unguided de-escalation reduce bleeding without any trade-off in ischemic events (53). While patients from these trials were mostly East Asians and underwent non-complex PCI, it is important to note that this strategy may not sufficiently consider the increased ischemic risk some subgroups of patients may exhibit, especially those with poor clopidogrel responsiveness and/or high ischemic risk. The ongoing VERONICA (NCT04654052) trial will provide important insights on the use of a PFT-guided de-escalation 1 month after ACS, combining the benefit of modulating P2Y_12_ inhibition after the highest risk period of thrombotic complications post-ACS is over with a personalized selection of P2Y_12_ inhibitors (Table 2).

**TABLE 2:** Ongoing studies on de-escalation strategies in acute coronary syndrome.

Study name	NCT	Strategy	Number of patients	Treatment arms and population	Primary endpoint
Optimized-APT	NCT04338919	Unguided modulation of P2Y_12_ inhibition	2,020	DAPT with ticagrelor 90 mg/bid for the first month, followed by ticagrelor 90 mg/bid monotherapy from the second to the sixth months and ticagrelor 45 mg/bid monotherapy from the sevenths to the twelfth months vs. DAPT with ticagrelor 90 mg/bid for 12 months	MACE NACE BARC 2–5 bleeding
ELECTRA-SIRIO	NCT04718025	Unguided modulation of P2Y_12_ inhibition	4,500	DAPT with ticagrelor 90 mg/bid for 1 month followed by DAPT with ticagrelor 60 up to 12 months vs. discontinuation of ticagrelor 60 mg/bid at 3 months vs. placebo	MACE BARC 3–5 bleeding
VERONICA	NCT04654052	PFT-guided de-escalation	634	Guided DAPT after 1 month vs. standard 12-month DAPT with prasugrel or ticagrelor	NACE
DUAL-ACS2	NCT03252249	Short DAPT followed by aspirin monotherapy	19,519	3-months vs. 12-month DAPT among ACS patients	All-death
TACSI	NCT03560310	Short DAPT followed by aspirin monotherapy	2,200	Ticagrelor and aspirin vs. aspirin monotherapy after isolated coronary artery bypass grafting	MACE
ELECTRA-SIRIO	NCT04718025	Short DAPT followed by a P2Y12 inhibitor monotherapy	4,500	DAPT with ticagrelor 90 mg/bid for 1 month followed by DAPT with ticagrelor 60 up to 12 months vs. discontinuation of ticagrelor 60 mg/bid at 3 months vs. placebo	MACE BARC 3–5 bleeding
NEO-MINDSET	NCT04360720	Short DAPT followed by a P2Y12 inhibitor monotherapy	3,400	Prasugrel monotherapy for 12 months vs. 12-month DAPT among non-HBR and ticagrelor monotherapy for 12 months vs. 6 months DAPT among HBR patients	MACCE and BARC bleeding 2–5
ULTIMATE-DAPT	NCT03971500	Short DAPT followed by a P2Y12 inhibitor monotherapy	3,486	Ticagrelor monotherapy after 1 month DAPT vs. standard DAPT	MACCE and BARC bleeding 2–5
CAGEFREEII	NCT04971356	Short DAPT followed by a P2Y12 inhibitor monotherapy	1,908	Aspirin plus ticagrelor for 1 month followed by 5 months ticagrelor monotherapy vs. aspirin plus ticagrelor for 12 months in patients with drug-coated balloon	NACE
BULK-STEMI	NCT04570345	Short DAPT followed by a P2Y12 inhibitor monotherapy	1,002	3-months DAPT followed by ticagrelor monotherapy vs. 12-months DAPT after second generation sirolimus stent	NACE
LEGACY	NCT05125276	Short DAPT followed by a P2Y12 inhibitor monotherapy	3,090	Ticagrelor monotherapy vs. standard DAPT	BARC bleeding 2–5

DAPT, dual antiplatelet therapy; PFT, platelet function test, BARC, Bleeding Academic Research Consortium; MACCE, major adverse cardiovascular and cerebrovascular events; NACE, net adverse clinical events; NCT, National Clinical Trial number.

## De-escalation: When?

**FIGURE 3:**
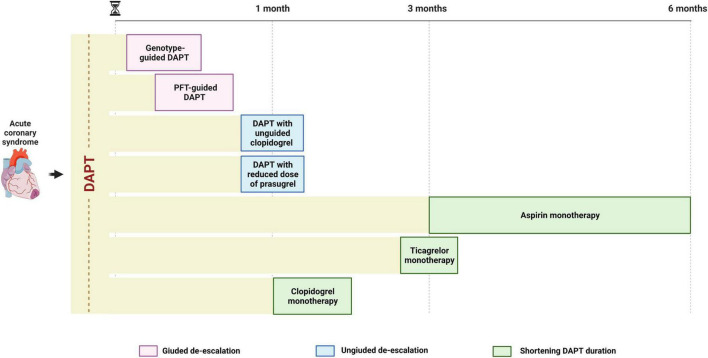
Timing for de-escalating antiplatelet therapy after ACS undergoing PCI. A guided de-escalation strategy allows for an early (days or weeks) de-escalation of antiplatelet therapy while unguided de-escalation is to be performed at least 1 month after ACS and shortening of DAPT is to be performed 1–6 months after ACS, depending on modality (aspirin or P2Y_12_ inhibitor monotherapy). ACS, acute coronary syndrome; DAPT, dual antiplatelet therapy; PCI, percutaneous coronary intervention.

Strategies of de-escalation of antiplatelet therapy may be implemented at different time points: 2–3 days post-PCI (i.e., guided de-escalation); after 1–3 months of DAPT (i.e., ticagrelor monotherapy); after 3–6 months of DAPT (i.e., aspirin monotherapy) (Figure 3). Because the number needed to treat to prevent a bleeding event is expected to be smaller in high bleeding risk (HBR) patients compared to the general population, such strategy may be particularly advantageous in this cohort of patients. Indeed, current guidelines recommend the use of clopidogrel over prasugrel and ticagrelor and the shortening of DAPT duration up to 1 or 3 months after PCI in non-ST elevation ACS (NSTE-ACS) and up to 6 months in ST-elevation ACS (STE-ACS) (7, 54) in HBR patients. A standardized definition of HBR is of essence for the prompt identification of such patients. In this regard, the Academic Research Consortium for HBR (ARC-HBR) definition including 14 major and 6 minor criteria represents the reference definition and defines as HBR patients with a BARC 3 or 5 bleeding risk of ≥ 4% at 1 year or a risk of an intracranial hemorrhage (ICH) of ≥ 1% at 1 year (55). Nevertheless, a de-escalation of antithrombotic therapy has been shown to be advantageous also among patients without HBR. Indeed, current guidelines recommend the use of ticagrelor monotherapy after 3 months of standard DAPT as an alternative to a standard 12 month-DAPT, with a class IIa, LOE B recommendation (54). Although guided and unguided de-escalation have shown encouraging results, important evidence in support of its use came after the publication of the most recent guidelines that currently recommend a de-escalation of P2Y_12_ inhibitor only for selected clinical scenarios, with a class IIb, LOE B recommendation (17, 54).

It is important to note that the effectiveness of a de-escalation strategy may be influenced by a number of factors that should be taken into account when adopting such a strategy. Indeed, clinical, demographics and sex-related characteristics may significantly impact the response to antiplatelets (33). In particular, Asian patients have been shown to be more susceptible to bleeding rather than ischemic events compared with Caucasian patients, despite *CYP2C19* LoF alleles being more frequent in Asian ancestry population, a condition known as the “Asian paradox” (33). Furthermore, recent data suggest woman may benefit the most from a de-escalation strategy consisting in P2Y_12_ inhibitor monotherapy after a short course of DAPT suggesting the need for understanding potential sex-specific response to antiplatelet therapy (56). It is worth noting that a substantial proportion of treated patients present individual characteristics such as diabetes mellitus, chronic kidney disease, obesity or advanced age that are associated with platelet hyperreactivity and increased thrombotic risk (57–64). Moreover, among patients undergoing PCI, procedural characteristics such as double stenting of coronary bifurcations, stenting of chronic total occlusion or long lesions requiring multiple stents are associated with an increased risk of ischemic recurrences (65). Because this enhanced ischemic risk may be partially overcome by more potent platelet inhibition, the use of a standard 12-month DAPT with prasugrel or ticagrelor, including prolongation of a more intense antithrombotic regimen beyond 12 months after ACS, should be considered over a de-escalation strategy particularly among low bleeding risk patients (65). Recent guidelines provide a number of clinical and procedural features as well as suggest the implementation of scores and definitions such as the PRECISE-DAPT score and the Academic Research Council definition of HBR for the stratification of bleeding and ischemic risk in patients with ACS (22, 62, 63).

## Future perspectives and conclusions

**FIGURE 4:**
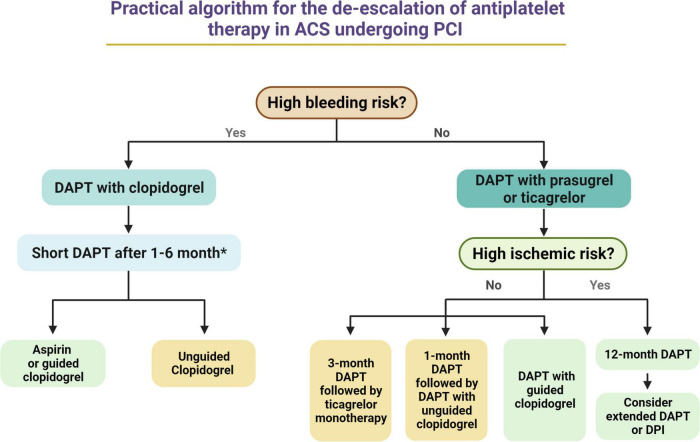
Practical algorithm for the de-escalation of antiplatelet therapy in ACS undergoing PCI. *Among HBR patients, DAPT duration may range from 1 month (i.e., low ischemic risk NSTEMI patients) to 6 months (i.e., high ischemic risk STEMI patients). Green Box, first choice. Yellow Box, second choice. ACS, acute coronary syndrome; DAPT, dual antiplatelet therapy; PCI, percutaneous coronary intervention; STEMI, ST-segment elevation myocardial infarction; NSTEMI, non-ST elevation myocardial infarction.

A de-escalation strategy of antiplatelet therapy represents a very promising strategy for reducing bleeding without any trade-off in ischemic events in patients with ACS. Nevertheless, available evidence present some limitations, such as the fact many studies have been performed on Asian populations, limiting the generalization of their results to other ethnicities as well as the fact they often used non-inferiority designs with primary endpoints including both bleeding and ischemic events, leading to reduced statistical power for hard ischemic and bleeding events. Despite pooled analysis have played a role in overcoming some of these limitations, others still persists. The use of a de-escalation in lieu of a standard 12-months DAPT with potent P2Y_12_ inhibitors should always be considered after a careful assessment of individual bleeding and ischemic risks, and, possibly, of individual response to an antiplatelet agent. HBR patients and those responding to clopidogrel should hypothetically benefit the most from such strategy, while the use of a de-escalation strategy may be even potentially detrimental in patients at high ischemic and low bleeding risk or in those not responding to clopidogrel. To this extent, the implementation of tools allowing for a guided selection of antiplatelet therapy may play an important role. Figure 4 provides a practical algorithm for de-escalating antiplatelet therapy in patients with ACS undergoing PCI. Among patients with HBR, DAPT duration can be shortened up to 1 month in low ischemic risk NSTEMI patients and up to 6 months in high ischemic risk STEMI patients. After DAPT interruption, aspirin should be still preferred over clopidogrel in case of unguided use of clopidogrel, while guided clopiodogrel monotherapy may represent an equally (or potentially better) valuable alternative to aspirin. Among patients without HBR or high ischemic risk, the use of a guided selection of antiplatelet therapy may represent the most valuable and tailor-made approach, while unguided clopidogrel use after 1 month of standard DAPT and ticagrelor monotherapy after 3 months of standard DAPT represent alternative options. Finally, patients without HBR who are at high ischemic risk may benefit of a standard 12-month DAPT with prasugrel or ticagrelor, that can be further prolonged (i.e., beyond 12 months) with any of the approved antithrombotic regimens (i.e., prolonged DAPT or dual pathway inhibition with low dose of rivaroxaban). Ongoing studies will provide further insights on de-escalation on antiplatelet therapy in patients with ACS (Table 2).

## Author contributions

Both authors listed have made a substantial, direct, and intellectual contribution to the work, and approved it for publication.
